# Supplementary material for: Establishing an early warning event management system at Africa CDC
Source: PLOS Digit Health. 2024 Jul 8;3(7):e0000546. doi: 10.1371/journal.pdig.0000546 (PMC11230544; doi:10.1371/journal.pdig.0000546)
Supplement: Attachment — Submitted filename: Response to Reviewers_PDIG-D-23-00468.docx [file pdig.0000546.s001.docx]

Dear Managing Editor and Reviewers,

Thank you so much for this thoughtful review of our manuscript. We have gone through and revised the manuscript as suggested. Responses are listed after each reviewer comment and within the updated manuscript. We have additionally included two new figures to address the process and data flow as well as the development timeline. The timeline replaces the previous tables 1 and 7.

Please let us know if there are any additional comments or areas of clarification that we can provide.

All the best,

_____________________________________________________________

PDIG-D-23-00468
Establishing an early warning Event Management System at Africa CDC
PLOS Digital Health

Dear Dr. TETUH,

Thank you for submitting your manuscript to PLOS Digital Health. After careful consideration, we feel that it has merit but does not fully meet PLOS Digital Health's publication criteria as it currently stands. Therefore, we invite you to submit a revised version of the manuscript that addresses the points raised during the review process.

Please submit your revised manuscript within 60 days May 06 2024 11:59PM. If you will need more time than this to complete your revisions, please reply to this message or contact the journal office at [digitalhealth@plos.org](mailto:digitalhealth@plos.org). When you're ready to submit your revision, log on to <https://www.editorialmanager.com/pdig/> and select the 'Submissions Needing Revision' folder to locate your manuscript file.

Please include the following items when submitting your revised manuscript:
*       A rebuttal letter that responds to each point raised by the editor and reviewer(s). You should upload this letter as a separate file labeled 'Response to Reviewers'.
*       A marked-up copy of your manuscript that highlights changes made to the original version. You should upload this as a separate file labeled 'Revised Manuscript with Track Changes'.
*       An unmarked version of your revised paper without tracked changes. You should upload this as a separate file labeled 'Manuscript'.

If you would like to make changes to your financial disclosure, please include your updated statement in your cover letter. Guidelines for resubmitting your figure files are available below the reviewer comments at the end of this letter.

We look forward to receiving your revised manuscript.

Kind regards,

Gilles Guillot
Academic Editor
PLOS Digital Health

Journal Requirements:

Additional Editor Comments (if provided):

While the manuscript presents interesting material, the reviewers have identified many aspects on which it should be improved.
If you decide to submit a revised version, please address all the points raised.

[Note: HTML markup is below. Please do not edit.]

Reviewers' comments:

Reviewer's Responses to Questions

**Comments to the Author**
1. Does this manuscript meet PLOS Digital Health’s [publication criteria](https://journals.plos.org/digitalhealth/s/journal-information#loc-criteria-for-publication)? Is the manuscript technically sound, and do the data support the conclusions? The manuscript must describe methodologically and ethically rigorous research with conclusions that are appropriately drawn based on the data presented.

Reviewer #1: Yes
Reviewer #2: Yes
--------------------

2. Has the statistical analysis been performed appropriately and rigorously?

Reviewer #1: Yes
Reviewer #2: Yes
--------------------

3. Have the authors made all data underlying the findings in their manuscript fully available (please refer to the Data Availability Statement at the start of the manuscript PDF file)? The [PLOS Data policy](https://journals.plos.org/digitalhealth/s/data-availability) requires authors to make all data underlying the findings described in their manuscript fully available without restriction, with rare exception. The data should be provided as part of the manuscript or its supporting information, or deposited to a public repository. For example, in addition to summary statistics, the data points behind means, medians and variance measures should be available. If there are restrictions on publicly sharing data—e.g. participant privacy or use of data from a third party—those must be specified.

Reviewer #1: Yes
Reviewer #2: Yes
--------------------

4. Is the manuscript presented in an intelligible fashion and written in standard English? PLOS Digital Health does not copyedit accepted manuscripts, so the language in submitted articles must be clear, correct, and unambiguous. Any typographical or grammatical errors should be corrected at revision, so please note any specific errors here.

Reviewer #1: Yes
Reviewer #2: No
--------------------

5. Review Comments to the Author. Please use the space provided to explain your answers to the questions above. You may also include additional comments for the author, including concerns about dual publication, research ethics, or publication ethics. (Please upload your review as an attachment if it exceeds 20,000 characters)

Reviewer #1: This is an interesting article that documents a real world experience of conceptualizing an operationalizing an open source data platform for event-based surveillance. I have the following comments:

1) Article should be formatted according to the PLOS Digital Health submission guidelines. Please review them carefully and adhere to them.
Some examples -
a) one of the authors has mentioned their educational qualifications in the title page - this is incorrect
b) references should not be cited in superscript in the paper
c) references should be in Vancouver style
d) author summary should be included

We have updated the formatting, including the citations to match the PLoS submission guidelines.

2) Abstract is too short - it does not summarize the paper well. The journal has a 300 word limit for the abstract, currently it is only 131 words, so you have scope to expand it further.

Thank you for bringing our attention to this. We have expanded the abstract to include more key outcomes from the manuscript.

3) The paper, while well written, seems to be written in the style of a project report rather than a manuscript.
Some examples -
a) lines 55 to 57 "We describe the system developed and its use in conducting EBS at Africa CDC, as well as its early adoption in African countries" can be updated to "This article describes the system developed and its use in conducting EBS at Africa CDC, as well as its early adoption in African countries".

Updated this language
b) Lines 42 to 43 - "We realize COVID-19 is not going to be the last pandemic" can be replaced with "It is well understood that COVID-19 will not be the last pandemic" and you can also cite a reference to support this - <https://www.orfonline.org/research/the-covid19-pandemic-why-it-wont-be-the-last>

We updated the language to make more general

4) Lines 27 to 29 - "This is not unexpected given that around 80% of global biodiversity, including pathogen diversity, is found in tropical regions". Is there a reference for this bold statement? If yes, please cite.

We have removed this statement and replaced it with something more in alignment and specific with the region.

5) The difference between "signal" and "event" is not clearly delineated.

Definitions for these are now included in the methods

6) Can the authors develop a figure to depict the workflow of the system and how it helps to facilitate EBS? How is data for an event entered into the system - manually by the team of users, or automatically from EIOS and other sources, or a mix of both options? What happens after the event is created ("detected") in the system? How is it verified? How is its risk assessed? how is it notified? How is the response initiated? This would be very helpful to ensure clarity for the reader.

We have developed the following figure to help depict the workflow of EBS and how it relates to the EMS.

7)  Line 131 - "Small consumable modules were released for pilot testing at monthly intervals". What does this mean? please clarify.

Updated to state, “Small iterative updates were….” To help clarify the intent

8) Lines 162 to 163 - "Switching from weekly to daily reporting for such major epidemics/pandemics is seamless." This seems like you are praising the system. Kindly do not use such language in an academic paper. Instead, you can rephrase it as - "The DHIS2 software enabled a seamless shift from weekly to daily reporting for COVID-19".

Updated this section to reflect the suggested wording.

9) Lines 172 to 173 - "In order to automatically calculate reporting statistics, worldwide population estimates per country, gender and age group were referenced from UN databases, and updated annually". Is this referring to the World Population Prospects which is annually published by the United Nations Population Division? If yes, please mention it and cite it as a reference (<https://population.un.org/wpp/>).

Updated to include

10) Lines 183 to 184 - "Other important custom functionality added included the addition of Africa CDC’s corporate colours to DHIS2..." Is the addition of Africa CDC's corporate colors to DHIS2 an important custom functionality? I would disagree and suggest that this be deleted. The other custom functionality, though, is important and should be retained (integration of EMS with BI platforms).

Updated this section as suggested

11) Please do a thorough review of the paper to eliminate errors in grammar and spelling.
Examples -
a) Line 215 - it should be "Eswatini", not "Estwatini".

Eswatini was not originally mentioned in this manuscript – we search for this possible reference and were unable to locate this
b) Line 237 - the last word should be "for", not "of".

We were also unable to locate this recommendation. To ensure that all spelling and grammar issues were addressed, we reran a full spell and grammar check and individually proofed the document one last time.

12) Lines 280 to 282 - "Given that Africa CDC has set a goal to establish EBS, possibly using a variant of the EMS, in most AU Member States by 2030, it will be important to be aware of and supportive of other EMSs established in Africa". Suggest replacing "supportive of" with "in alignment with".

Updated to reflect this new language

13) Lines 290 to 291 - "The EMS provides a solution not previously available and is something readily available to other global regions and countries" - the meaning of this sentence is unclear, please review.

We have updated this section to now read: The EMS provides an EBS data management solution not previously available that can be easily adapted to support other global regions and countries. It is in alignment with and supports Africa CDC’s effort to strengthen data standards and data sharing across Africa’s disease surveillance community, and the system is expected to be steadily improved over the coming years.

14) Lines 299 to 302 - I do not think this section is needed "Biography of first author", please delete it.

removed

15) Please include an acknowledgements section - the efforts of the EMS users and the HISP developers and the country teams where EMS piloting is occurring need to be acknowledged.

added

16) Table 1 - For the May 2022 row, the description is "See framework M&E". What does this mean? Is there an annexe for framework M&E?

we have updated this and added a citation to the mentioned framework

17) In Table 3 and Table 6, the total number of events that occurred in 2020, 2021 and 2022 are 93, 126 and 197 respectively. But in Table 5, they are 93, 131 and 208. How can these totals be different? Please review and reconcile these differences.

Table 3 (now table 2) references country-level events, whereas table 5 (now table 4) also includes signals, or unverified events. We have updated the title of table 5 (now table 4) to reflect this additional data.

18) What is the source of funding for the EMS? Please mention it in the Introduction section in lines 53 to 55 -

We have added the following: "In response to this need, Africa CDC in collaboration with HISP-SA developed an event management system (EMS) with initial funding from the African Union to support the implementation of EBS at the continental level but also provide a helpful solution to countries interested in improving EBS related workflow."

19) Is there a sustainability plan in place to ensure the hosting and routine maintenance of the EMS even after cessation of donor funding? Please include a sentence about that in the Discussion section in the paragraph on next steps.

Following the development of the system, HISP-SA conducted a knowledge transfer to the Africa CDC system managers. Given the system is open source, countries have set up country teams with expertise in managing the DHIS-2. These teams are now supporting country customization and maintenance. We have added language addressing these aspects to the discussion section.

Reviewer #2: The manuscript is generally well written, and I would advise the manuscript to be published.

One thing that should be improved is that the manuscript focuses mainly on the anecdotal story of how the system was set up and spends little time on the actual results of the system (tables 4, 5, and 6). In fact, only two paragraphs are focussed on this key part of the manuscript.

Also, I do believe that the manuscript should focus slightly more on how the system has supported early detection. The manuscript doesn't have any metric to understand how early detection was improved by using this system. For example, where are these alerts reported by other systems from member states? If the data for this is not available, which I understand can be difficult to source, a discussion section could be added to explain this.

The event management system serves as a platform to improve the EBS workflow. Early detection may not be attributable to the actual system, but more so the actual modality of EBS. A robust evaluation of EBS, comparing the sensitivity of EBS as a surveillance modality to another surveillance modality (e.g., IBS) would need to be completed to show this aspect about detection. This was not the intent of this manuscript – however we were still able to measure some aspects of timeliness and improvement on this over time.

A few other points:
Abstract: -information provided by the abstract does not support the last sentence of the abstract
-You mention countries adapting, do you mean adopting?

We have updated the abstract to reflect the term adaptation

Introduction:
line 26. Mention the number of PHEIC in Africa

Updated
Line 38: please refrain from qualifiers when referring to 'astute' epidemiologists

Removed term
Line 42: please refrain from personal opinions and focus on facts or rephrase it so that is not an opinion

This was rephrased to state that it is well understood with an added citation.
Line 49: here you introduce the EBS at the ACDC. Please introduce it before

EBS, specifically media scanning at Africa CDC was noted in the second paragraph, “The Africa Centres for Disease Control and Prevention (Africa CDC) specifically used the EBS modality of media scanning to monitor the spread of COVID-19 globally as well as the introduction and progression in Africa.” Please let us know if this is not a sufficient mention.
Line 53: spell out HISP-SA

Updated
Line 104-105. I'm unsure this information is relevant to the reader

Removed
Line 163: seamless doesn't seem to be a measurable metric

Removed sentence
Line 183-184: If I understand correctly, here you mention the colour of the dashboard? If so, please remove as this is likely not relevant to EWARN implementation

Removed and further clarified the following sentences
Table 2. 'Created de novo' please rephrase to created

Updated
Table 5: the last two indicators are not clear on what an intervention by ACDC or a country is. Please define

An intervention would be a response related activity. We have added a definition for this in the methods section.
Table 6: indicator 4. Notified to who? Indicator 5: what constitutes a response?

Updated this language to reflect the exact action that was taken

--------------------

6. PLOS authors have the option to publish the peer review history of their article [what does this mean?](https://journals.plos.org/digitalhealth/s/editorial-and-peer-review-process#loc-peer-review-history) If published, this will include your full peer review and any attached files.

Do you want your identity to be public for this peer review? If you choose “no”, your identity will remain anonymous but your review may still be made public.

For information about this choice, including consent withdrawal, please see our [Privacy Policy](https://www.plos.org/privacy-policy)

Reviewer #1: No
Reviewer #2: No
--------------------


[NOTE: If reviewer comments were submitted as an attachment file, they will be attached to this email and accessible via the submission site. Please log into your account, locate the manuscript record, and check for the action link "View Attachments". If this link does not appear, there are no attachment files.]

While revising your submission, please upload your figure files to the Preflight Analysis and Conversion Engine (PACE) digital diagnostic tool, <https://pacev2.apexcovantage.com/>. PACE helps ensure that figures meet PLOS requirements. To use PACE, you must first register as a user. Registration is free. Then, login and navigate to the UPLOAD tab, where you will find detailed instructions on how to use the tool. If you encounter any issues or have any questions when using PACE, please email PLOS at [figures@plos.org](mailto:figures@plos.org). Please note that Supporting Information files do not need this step.

__________________________________________________
In compliance with data protection regulations, you may request that we remove your personal registration details at any time.  (Use the following URL: <https://www.editorialmanager.com/pdig/login.asp?a=r>). Please contact the publication office if you have any questions.
