# Supplementary material for: Establishing an early warning event management system at Africa CDC
Source: PLOS Digit Health. 2024 Jul 8;3(7):e0000546. doi: 10.1371/journal.pdig.0000546 (PMC11230544; doi:10.1371/journal.pdig.0000546)
Supplement: Attachment — Submitted filename: Response to Reviewers_PDIG-D-23-00468 5June.docx [file pdig.0000546.s002.docx]

Dear Managing Editor and Reviewers,

Thank you so much for this thoughtful review of our manuscript. We have gone through and revised the manuscript as suggested. Responses are listed after each reviewer comment and within the updated manuscript.

Please let us know if there are any additional comments or areas of clarification that we can provide.

All the best,

Editorial Requirements for Revisions

1. Please review your reference list to ensure that it is complete and correct. If you have cited papers that have been retracted, please include the rationale for doing so in the manuscript text, or remove these references and replace them with relevant current references. Any changes to the reference list should be mentioned in the rebuttal letter that accompanies your revised manuscript. If you need to cite a retracted article, indicate the article's retracted status in the References list and also include a citation and full reference for the retraction notice.

We have reviewed our references and fix the style but no cited paper was retracted.

2. Please provide separate figure files in .tif or .eps format only and remove any figures embedded in your manuscript file. Please also ensure that all files are under our size limit of 10MB.

This has been done and all figures have been separately attached.

Additional Editor Comments (if provided):

In many cases (introduction) the citation style used by the authors is of the form
(ref number) Claim....
This is unsual.
The common usage is Claim.... (ref number).

As requested earlier by one of the reviewer: please check the journal citation style and submit a revision that complies with it.
Handling this type of formatting issue is the responsibility of the authors.
It was not appropriate to rely on the reviewers to perform this task.

We have fix this throughout the manuscript

Abstract:
- Sentence "DHIS2 was identified as the platform for
the EMS as it can support data capture, monitoring, analysis, and reporting of events." is unclear if one is not familiar with DHIS2

DHIS2 written in full and the following sentence included to provide clarity. “The district health information software (DHIS2), which is free and open-source software…..”

- Spell out EBS in its first occurence (abstract)

EBS changed to “event-based surveillance” in the abstract
